# Direct costs of radiotherapy for rectal cancer: a microcosting study

**DOI:** 10.1186/s12913-015-0845-9

**Published:** 2015-05-02

**Authors:** Paul Hanly, Alan Ó Céilleachair, Máiréad Skally, Ciaran O’Neill, Linda Sharp

**Affiliations:** School of Business, National College of Ireland, Dublin, Ireland; Research Department, National Cancer Registry Ireland, Cork, Ireland; Department of Clinical Microbiology, Beaumont Hospital, Dublin, Ireland; J.E. Cairnes School of Business and Economics, National University of Ireland, Galway, Ireland

**Keywords:** Radiotherapy, Microcosting, Rectal cancer, Efficiency

## Abstract

**Background:**

Radiotherapy provides significant benefits in terms of reducing risk of local recurrence and death from rectal cancer. Despite this, up-to-date cost estimates for radiotherapy are lacking, potentially inhibiting policy and decision-making. Our objective was to generate an up-to-date estimate of the cost of traditional radiotherapy for rectal cancer and model the impact of a range of potential efficiency improvements.

**Methods:**

Microcosting methods were used to estimate total direct radiotherapy costs for long- (assumed at 45-50 Gy in 25 daily fractions over a 5 week period) and short-courses (assumed at 25 Gy in 5 daily fractions over a one week period). Following interviews and on-site visits to radiotherapy departments in two designated cancer centers, a radiotherapy care pathway for a typical rectal cancer patient was developed. Total direct costs were derived by applying fixed and variable unit costs to resource use within each care phase. Costs included labor, capital, consumables and overheads. Sensitivity analyses were performed.

**Results:**

Radiotherapy treatment was estimated to cost between €2,080 (5-fraction course) and €3,609 (25-fraction course) for an average patient in 2012. Costs were highest in the treatment planning phase for the short-course (€1,217; 58% of total costs), but highest in the radiation treatment phase for the long-course (€1,974: 60% of total costs). By simultaneously varying treatment time, capacity utilization rates and linear accelerator staff numbers, the base cost fell by 20% for 5-fractions: (€1,660) and 35% for 25-fractions: (€2,354).

**Conclusions:**

Traditional radiotherapy for rectal cancer is relatively inexpensive. Moreover, significant savings may be achievable through service organization and provision changes. These results suggest that a strong economic argument can be made for expanding the use of radiotherapy in rectal cancer treatment.

## Background

Colorectal cancer is the third most commonly diagnosed cancer worldwide with over one million new cases annually [1]. Around one-third of these cases arise in the rectum. Radiotherapy has been shown to provide significant benefits in terms of reducing risk of local recurrence and death from rectal cancer [2]. Despite this, there is evidence that radiotherapy may not be given to as many patients as could benefit. An Australian study estimated that more than half of patients with newly diagnosed cancers could benefit from radiotherapy [3]. Internationally, actual utilization rates have been shown as lower than this [4-7].

Although underutilized, conventional radiotherapy for rectal cancer appears to be relatively inexpensive. Five-year costs for diagnosis and management of a case of rectal cancer has been estimated to be approximately €43,000 [8]. In 2008, a course of conventional radiotherapy for rectal cancer cost was estimated to cost €3,239 [9]. However, this cost was obtained from reviewing studies from a range of jurisdictions, most of which were undertaken before or around 2000: no more up-to-date estimates appear to be available. This lack of up-to-date cost estimates potentially inhibits policy and decision-making, and hinders cost-effectiveness comparisons with various novel radiotherapy treatment strategies which have been recently developed [10].

We used microcosting methods to generate an up-to-date estimate of the economic cost of traditional radiotherapy treatment for rectal cancer. We also modeled the impact on costs of a range of potential efficiency improvements in the provision of radiotherapy.

## Methods

The microcosting methodology is a technique that attempts to enumerate and cost all inputs consumed due to a medical intervention. The method purports to accurately reflect actual economic costs associated with an intervention and can provide a key input for undertaking economic evaluations. Using this technique we calculated total direct costs for an average short-course (assumed at 25 Gy in 5 daily fractions over a one week period) and an average long-course (assumed at 45-50 Gy in 25 daily fractions over a 5 week period) of radiotherapy for rectal cancer across different phases of care (as outlined below). Costs are reported from the perspective of the radiotherapy department in 2012 euros.

### Setting

The study was conducted in Ireland, which has a mixed public-private healthcare system. All citizens are entitled to treatment in the public system and the majority make modest co-payments for overnight hospital stays or outpatient appointments. Public cancer services are provided in eight designated cancer centers which operate within Managed Cancer Control Networks.

### Ethics statement

This study has been approved by the appropriate hospital ethics committees. No identifiable human data were used for this study.

### Data collection and phases of care

Data collection involved structured interviews by two of the authors with senior management and clinical staff in the radiotherapy units of two designated cancer centers during 2010 and 2011. In total, three interviews were undertaken with: a radiation oncologist, a radiotherapy services manager and a colorectal nurse specialist. The interview schedule was devised from review of the microcosting literature [11-15] and previous author interviews with colorectal cancer clinicians (which focused on treatment options). On-site direct observation was also undertaken supplemented by follow-up discussions with the radiology team to appraise any additional items that required clarification. On-site observation included visual confirmation of personnel in attendance during treatment, appraisal of capital equipment employed, and confirmation of consumables used.

A pathway of care for an “average” (typical) rectal cancer patient was subsequently developed encompassing three “phases”: treatment planning; radiation treatment; and follow-up.

### Cost calculation

Total direct costs were derived by applying unit costs to resource use within each phase of care. Resource use was identified in natural units (Table 1) across four broad components: labor (time), capital (equipment and maintenance), consumables and overheads. To compute total costs, we aggregated ‘fixed’ planning and follow-up costs (these costs did not vary on a fraction basis and thus were considered ‘fixed’) and ‘variable’ treatment costs (per fraction) for long- and short-course radiotherapy treatment. Estimates for a 21 fractions course are also provided for completeness. For example, the fixed cost for a long course of treatment was €1,217.2. The variable treatment cost was €90.9 per fraction. Over a long course this amounted to €2,272.5 (€90.9*25). Follow up costs were a fixed sum of €119.2 per patient. The aggregated total of the three care phases for 25 fractions was €3,608.9.

**Table 1:** **Unit costs (€ 2012) for radiotherapy care resource use by phase of care (25 fraction course)**

**Phase**	**Cost category**	**Description**	**No. of units**	**Cost per unit per minute (€)**	**Cost per procedure (€)**
**Treatment planning**	***Capital***	CT simulation and linear accelerator	1	2.10	62.9
		Moulding equipment	1	0.26	7.9
	***Salary***	Radiation therapist	2	0.44	26.2
		Clinical specialist radiographer	2	0.54	32.4
		Dosimetrist	2	0.61	285.1
		Physicist	1	0.52	31.3
		Staff nurse	1	0.37	22.3
		Radiation oncologist	1	1.65	66.0
		Dietician	1	0.43	12.9
	***Overheads***	Simulation	-	-	23.4
		Care plan	-	-	126.5
		Medical work-up	-	-	47.5
	***Other***				
		Blood test	-	-	17.0
		Additional imaging	-	-	438.0
**Treatment**	***Capital***	Linear accelerator	1	2.10	31.4
		Moulding equipment	1	0.26	3.0
	***Salary***	Radiation therapist	2	0.44	13.1
		Clinical specialist radiographer	2	0.54	16.2
		Radiation oncologist/Registrar	1	1.10	3.3
	***Overheads***	General	-	-	13.0
	***Consumables***	Fixation devices or shielding blocks for a proportion of patients	-	-	10.9
**Follow-up**	***Salary***	Radiation oncologist/Registrar	-	1.10	22.1
	***Overheads***	General	-	-	8.8

Labor costs included the time allocated to radiotherapy services by all relevant personnel. Time costs were calculated on a per minute basis initially, and then multiplied by the time allocated to each procedure for an average patient to determine per procedure costs. Salary scales per annum for relevant personnel was converted to a per minute rate by dividing by the number of workable minutes per year. We assumed a 48 week working year and a 35 hour working week for all staff with the exception of radiation oncologists (where we assumed a 37 hour working week). In addition consumables included the costs of fixation devices or shielding blocks for certain patients.

The cost of capital was annuitized over its expected useful economic life using the equivalent annual cost method [16]. Purchase prices for equipment were obtained from the two hospitals and anticipated life expectancy was set at 10 years. Equipment was assumed to operate at 100% capacity and costs were discounted at 4% per annum [17]. An estimate for molding equipment was taken from Kesteloot, Lievens, and van der Schueren [12] and updated to current prices, using an Irish health CPI (Central Statistics Office Ireland). Equivalent annual costs were divided by the number of workable minutes per year (assuming a 50 week working year and a 40 hour working week) to derive per minute equipment costs. Per procedure costs were estimated similar to labor costs. Equipment repair and maintenance costs were based on hospital budget costs. Building costs were not included.

Overheads were valued at 40% of the total direct salary of each relevant staff member following Irish Health Technology Assessment Guidelines [17]. These costs were assumed to cover accommodation, utilities (light, heat and telephone), support and back-office staff and training.

### Efficiency and sensitivity analyses

In sensitivity analyses we varied the following parameters in a univariate fashion based on suggestions made by interviewees: time per radiotherapy session, capacity utilization rates, linear accelerator staff numbers and overheads. The potential for efficiency improvements was assessed by simultaneously altering three parameters as follows: treatment time of 10 minutes compared to 15 minutes in current practice, capacity utilization rates of 125% (based on proposals for extensions to the standard working day of radiotherapy units) compared to 100% of the current working day for labor and capital and three linear accelerator staff compared to the current practice of four.

## Results

### Resource use and per minute costs

Resource use and unit costs for each cost category in each phase of care is presented in Table 1. During the treatment planning phase, the largest single cost item on a per procedure basis was the cost of dosimetrist services (€285). The highest cost per procedure in the treatment phase was the cost of the linear accelerator (€31.50). Labor costs dominated costs in the follow-up phase.

### Radiotherapy costs per fraction

In total, the ‘fixed’ treatment planning costs for a course of radiotherapy amounted to €1,217 (Table 2). Labor was the largest driver accounting for 41% of this total, with much of the labor cost generated by dosimetrist services. Other imaging services and tests accounted for a further 37% of total costs, with overheads accounting for 16% and capital for 6%.

**Table 2:** **Per patient and per fraction radiotherapy treatment (25 fractions) costs (€ 2012)**

**Type**	**Treatment planning**	**%**	**Treatment**	**%**	**Follow-up**	**%**
	Fixed costs: Cost (€) per patient*		**Variable costs: Cost (€) per fraction per patient**		Fixed costs: Cost (€) per patient*	
Capital	62.9	5.8	34.4	38.5	0.0	0.0
Labour	493.6	40.6	32.6	35.5	66.3	71.4
Overheads	197.4	16.2	13.0	14.2	26.5	28.6
Other/consumables	455.3	37.4	10.9	11.8	0.0	0.0
**Total**	**1,217.2**		**90.9**		**92.8**	

*Treatment planning costs and follow-up costs are the same irrespective of the length of course.

Treatment costs per fraction were estimated at €92 per patient per 25 fraction course (Table 2), €94 per patient per 21 fraction course and €154 per patient per 5 fraction course. Capital (35%) and labor (33%) generated over two-thirds of the 25 fraction total. The proportion of total costs accounted for by capital and labor for a 5 fraction course is somewhat less (57%).

In the follow-up phase, total ‘fixed’ costs were €93 per patient, with more than 70% of this accounted for by labor costs and the remainder by overheads.

### Radiotherapy costs per short- and long-course

A long-course of radiotherapy treatment was estimated to cost €3,609 for 25 fractions (€3,284 for 21 fractions). For an average 25 fraction patient, the majority of costs were accrued in the treatment phase (60%); 37% of costs were accrued in the planning phase and the remaining 3% during follow-up. Short-course estimates (5 fractions) were €2,080. In this instance, planning phase costs dominated accounting for 59% of the total.

### Sensitivity and efficiency analyses

**Figure 1:**
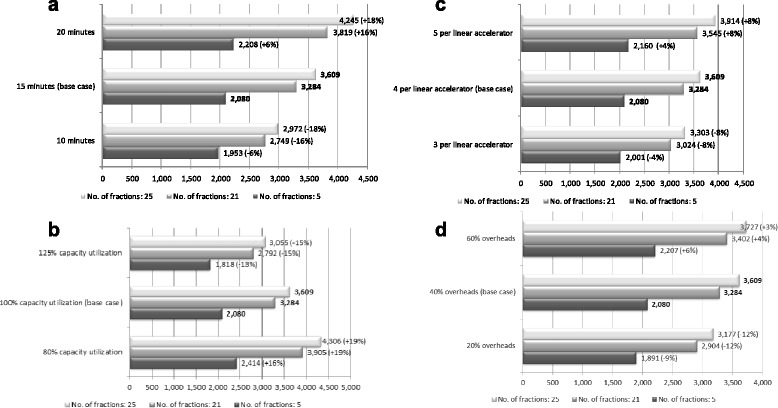
Sensitivity analysis of key radiotherapy cost drivers: total costs per course and percentage deviation from base case estimates (in brackets), by number of fractions per course (2012€). **a**. variations in treatment time (Treatment time refers to the time taken per radiotherapy session per procedure. 15 minutes relates to current practice). **b**. variations in capacity utilization (Capacity utilization refers to the time both labor and capital operate in the radiotherapy department on a given day. 100% relates to current working hours). **c**. variations in staff numbers (Staff numbers refers to the number of personnel per linear accelerator per procedure. Four staff members per linear accelerator relates to current practice). **d**. variations in overheads (Overheads refers to the costs of accommodation, utilities (light, heat and telephone), support and back-office staff and training for radiotherapy. 40% relates to Irish current recommendations on the costing of overheads in relation to direct salary costs).

Varying treatment time from 15 minutes (base case) to 10 minutes caused the total cost per course to fall by 6% (5 fractions) - 18% (25 fractions); when it was increased to 20 minutes, the total cost rose by between 6% and 18% (Figure 1a). Changing capacity utilization from 100% to 80% caused the base case estimate to increase by 16%-19%; increasing utilization to 125% reduced the total cost by 13-15% across courses (Figure 1b). The total radiotherapy cost was relatively insensitive to changes in linear accelerator staff numbers and overhead costs (Figure 1c and d).

**Figure 2:**
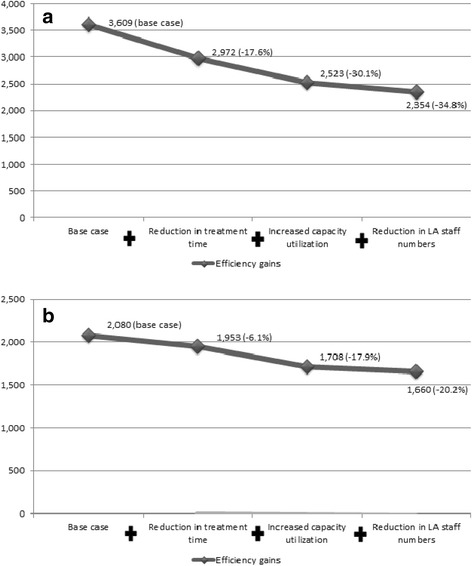
The impact of efficiency gains on the costs of radiotherapy (€) with percentage deviation from base case costs in brackets. **a**. 25 fraction course (Each nodal point represents the accumulated cost reduction resulting from the previous efficiency gains). **b**. 5 fraction course.

Combined incremental variation of treatment time (to 10 minutes), capacity utilization (to 125%) and linear accelerator staff numbers (to 3) resulted in a decrease in the base case estimate of total costs for long-course radiotherapy (25 fractions) of 35%, from €3,609 to €2,354 (Figure 2a). For short-courses, these changes would reduce the estimate of total cost per course by 20%, from €2,080 to €1,660 (Figure 2b).

## Discussion

### Costing framework and transferability

Our study attempted to address some of the criticisms made of previous radiotherapy costing studies [9,18]. Previous rectal cancer radiotherapy cost estimates vary widely, and a lack of transparency in reporting makes it difficult to explain these differences [9]. We took a “survey-based” microcosting approach, which has been used successfully in the past for the costing of various healthcare interventions [15,19]. By interviewing department managers, clinicians and other staff, we were able to define a typical care pathway for an average patient. Each separate process and resource within the pathway was delineated and apportioned a unit cost. This approach provided a clear framework for presenting results and the separate reporting of unit costs should further increase the comparability of the study [15]. The presentation of unit costs also ensures that estimates can be altered with country-specific data in other settings. Costs per fraction, and costs per course, were presented independently to allow future investigators, policy makers or planners to use or synthesis these as required. Overall, this standardized framework should enhance the transferability of our results to different settings.

### Radiotherapy treatment costs and components

The average cost per course in 2012 was €2,080, €3,284 and €3,609 for 5, 21 and 25 fractions respectively. Our estimate for a 21 fraction course is slightly higher than the average cost calculated by Ploquin and Dunscombe [9] (mean normalized cost in 2005, €3,239). It is impossible to be entirely certain what costs were included and excluded from previous studies due to a paucity of presented detail, however comparisons across broad cost components can be made.

Unsurprisingly, in our study, labor costs were a key cost driver, accounting for between 36% and 41% of total costs incurred during the planning and treatment phases. This result is relatively consistent throughout the radiotherapy costing literature where labor costs commonly account for roughly half of the costs of radiotherapy treatment [12,14]. Capital costs represented 26% of the cost for a 25 fraction course and were most significant during the treatment phase, consistent with the findings of previous radiotherapy costing studies [12,14]. Where our results differed from others was that overheads accounted for a higher proportion of total costs in our study. Although recommended in Ireland [17] the derivation of overhead costs based on a specific percentage of labor costs is not standard practice in other countries and may account for the difference. Nevertheless, the total cost estimate was relatively insensitive to variations in overheads.

### Potential cost savings

Through “efficiency analyses”, informed by expert opinion from our interviews, we were able to highlight key process areas in which efficiencies might be accrued by changing current service provision practices. These areas included: time taken per radiotherapy treatment procedure, capital utilization rates and the number of staff required per linear accelerator. While our results relate to practice in Ireland, similar service provision changes have been proposed in other healthcare systems.

Initially we assumed that an average treatment session could be cut from 15 to 10 minutes, resulting in a reduction of almost one-fifth in the total cost of long-course radiotherapy. An estimate of 10 minutes is consistent with best practice in Irish cancer centers (interview finding). It is however, possible that reducing procedure time could impact negatively on quality or, patient satisfaction, or increase the proportion of complicated cases, but we are not aware of any evidence in this regard.

A further amendment that has been discussed in Ireland is the extension of service hours. To reflect this, we assumed an increase in capacity of 25%, which effectively represents an extension of radiotherapy department operating hours from 9 am–5 pm to 8 am–6 pm. This change alone resulted in a reduction in total cost per course of up to one-quarter. However extension of the treatment day possesses both advantages and disadvantages which have been investigated across Irish, Dutch and UK radiotherapy treatment departments [20]. While increasing patient throughput and access, extended hours could have other consequences, including changes to shift work systems and flexible working arrangements, which may cause industrial relations issues. Health and safety issues would also require monitoring and there could be a knock-on effect on the working life of linear accelerators.

Currently, radiotherapy departments in Ireland operate with four personnel per linear accelerator similar to the staffing arrangements in the Netherlands, the UK and Australia [20]. Reducing the number to three could result in a reduction of costs of up to 9%. However, such a restructuring would run counter to current recommendations in the recent Report of the Expert Group on Radiography Grades in Ireland [21], and would require monitoring to ensure that standards are maintained and the quality of treatment does not suffer. A further potential efficiency improvement relates to the mix of staff present at a radiotherapy treatment session. The use of staff members at lower pay grades would result in lower labor costs. However as this measure was not suggested by any of our interviewees, we chose not to model it.

Cost savings between 20% (short-course) and 35% (long-course) were derived based on the incremental adoption of three potential efficiency enhancing changes. We would argue that these results represent feasible cost reductions across a range of areas in treatment provision and would further enhance the appeal of radiotherapy as a treatment option in rectal cancer. Nevertheless, key concerns such as potential industrial relations issues and impacts on quality would require further investigation (and clarification) before proceeding with these measures.

### Strengths and limitations

This study provides an up-to-date estimate of the cost of standard radiotherapy for rectal cancer which is of value in its own right for service planning and management and could be used as a comparator for evaluations of more novel radiotherapy approaches.

The study has several limitations. Data on resource use to populate the patient pathways were obtained from two public hospitals, but there is little reason to believe that these are not typical of the 8 designated cancer centers in Ireland. However, private hospital radiotherapy treatment provision may differ in terms of organization and practice. Our use of expert interviews and the compilation of resource use for an ‘average’ radiotherapy patient necessarily limits the detail in the results compared to, for example, a review of individual patient records or costs derived from the direct observation of actual resource utilization during treatment for individual patients. Nevertheless, using samples of patient records can lead to biases in the estimates due to outlier cases which often skew the results when the sample size is small [15], while direct observation is extremely resource intensive and normally requires additional supplementary consultation and/or interview with hospital departments to collect supplementary data. An overview of existing techniques for microcosting studies is provided by Frick.

Our sole focus was to estimate direct costs from the perspective of the radiotherapy department; we did not attempt to calculate indirect costs, or patient out-of-pocket costs. Our estimates do not include the capital costs of buildings, due to a lack of information available at the surveyed institutions. Furthermore, overhead costs were allocated based on a proportion of labor costs. Overhead costs, more than any other cost component, tends to be location and center specific and so their transferability is generally limited anyway. Finally we did not attempt to model the impact of patients who cancel appointments or do not attend for treatment.

## Conclusion

Using microcosting methods, we estimated that the total cost of traditional radiotherapy for rectal cancer in 2012 was €2,080 for an average short-course patient and €3,284 and €3,609 for an average long-course patient (21 fractions and 25 fractions respectively). Labor was a significant cost driver. Significant savings are potentially achievable through changes in service organization and provision. Given the effectiveness of radiotherapy in treating rectal cancer, and it’s relatively low cost compared to other cancer treatments, a strong argument can be made for expanding utilization of radiotherapy.

## References

[1] Ferlay J, Shin HR, Bray F, Forman D, Mathers C, Parkin DM (2010). Estimates of worldwide burden of cancer in 2008: GLOBOCAN 2008. Int J Cancer.

[2] Colorectal Cancer Collaborative Group (2001). Adjuvant radiotherapy for rectal cancer: A systematic overview of 8,507 patients from 22 randomised trials. Lancet.

[3] Delaney G, Jacob S, Featherstone C, Barton M (2005). The role of radiotherapy in cancer treatment: Estimating optimal utilization from a review of evidence-based clinical guidelines. Cancer.

[4] National Cancer Registry Ireland. Cancer in Ireland: Annual report of the National Cancer Registry. 2011. [http://www.ncri.ie/publications/annual-statistical-reports/cancer-ireland-2011]

[5] National Cancer Registry Ireland. Cancer trends no 9. Cancers of colon, rectosigmoid junction and rectum 2011. [http://www.ncri.ie/publications/cancer-trends-and-projections/cancer-trends-cancers-colon-and-rectum]

[6] Williams MV, Drinkwater KJ (2009). Radiotherapy in England in 2007: Modelled demand and audited activity. Clin Oncol (R Coll Radiol).

[7] Landrum MB, Keating NL, Lamont EB, Bozeman SR, McNeil BJ (2012). Reasons for underuse of recommended therapies for colorectal and lung cancer in the veterans health administration. Cancer.

[8] Tilson L, Sharp L, Usher C, Walsh C, Whyte S, O'Ceilleachair A (2012). Cost of care for colorectal cancer in Ireland: A health care payer perspective. Eur J Health Econ.

[9] Ploquin NP, Dunscombe PB (2008). The cost of radiation therapy. Radiother Oncol.

[10] Van de Werf E, Verstraete J, Lievens Y (2012). The cost of radiotherapy in a decade of technology evolution. Radiother Oncol.

[11] Dunscombe P, Roberts G, Walker J (1999). The cost of radiotherapy as a function of facility size and hours of operation. Br J Radiol.

[12] Kesteloot K, Lievens Y, van der Schueren E (2000). Improved management of radiotherapy departments through accurate cost data. Radiother Oncol.

[13] Lievens Y, van den Bogaert W, Kesteloot K (2003). Activity-based costing: A practical model for cost calculation in radiotherapy. Int J Radiat Oncol Biol Phys.

[14] Norlund A (2003). SBU Survey Group. Costs of radiotherapy Acta Oncol.

[15] Frick KD (2009). Microcosting quantity data collection methods. Med Care.

[16] Drummond M, Sculpher M, Torrance G, O'Brien, BJ, Stoddart, GL. Methods for the economic evaluation of healthcare programmes. 3rd Edn. Oxford University Press. 2005.

[17] Health Information and Quality Authority. Guidelines for the economic evaluation of health technologies in Ireland. 2010. [http://www.hiqa.ie/publication/guidelines-economic-evaluation-health-technologies-ireland]

[18] Goddard MK, Hutton J (1991). What is the cost of radiotherapy?. Eur J Radiol.

[19] Griffith GL, Tudor-Edwards R, Gray J, Butler R, Wilkinson C, Turner J et al. A micro costing of NHS cancer genetic services. Br J Cancer. 2005;92:60–71. 10.1038/sj.bjc.6602270 PMC2361743 15583691

[20] Department of Health. The development of radiation oncology services in Ireland. 2003. [http://www.hse.ie/eng/services/list/5/cancer/pubs/reports/Development_of_Radiation_Oncology_in_Ireland.pdf]

[21] Department of Health. Report of the expert group on radiography grades. July, 2001. [http://www.lenus.ie/hse/handle/10147/42528]

